# Immunohistochemical Analysis of Foxp3^+^, CD4^+^, CD8^+^ Cell Infiltrates and PD-L1 in Oral Squamous Cell Carcinoma

**DOI:** 10.1007/s12253-017-0270-y

**Published:** 2017-07-01

**Authors:** Olga Stasikowska-Kanicka, Małgorzata Wągrowska-Danilewicz, Marian Danilewicz

**Affiliations:** 1 Department of Nephropathology, Medical University of Lodz, ul. Czechoslowacka 8/10, 92-216 Lodz, Poland; 2 Department of Pathomorphology, Medical University of Lodz, Lodz, Poland

**Keywords:** Treg, FoxP3, PD-L1, Oral cancer

## Abstract

The immunoexpression of the PD-L1 and the number of immune infiltrating cells have been shown to be a significant prognostic factors in various human cancers. Immunohistochemical method was used to examine the immunoexpression of PD-L1 and number of Foxp3+, CD4+, CD8+ cells in 78 cases of oral squamous cell carcinomas (OSCCs): with better prognosis - OSCCBP (*n* = 37), and with poorer prognosis - OSCCPP (*n* = 41), and 18 cases of normal mucosa as a control. The immunoexpression of PD-L1 and the mean number of Foxp3+ cells was significantly increased in OSCCPP group in comparison to OSCCBP and control groups. The mean number of CD4+ cells was significantly increased in OSCCPP group in comparison to OSCCBP and control groups. CD8+ cells were significantly more numerous in OSCCBP group in comparison to OSCCPP and control group. In both OSCCPP and OSCCBP groups there were positive significant correlations between number of Foxp3+ and CD4+ cells. We found positive correlations between the immunoexpression of PD-L1 and numbers of Foxp3+ cells, and negative correlation between the immunoexpression of PD-L1 and numbers of CD8+ cells in both OSCCPP and OSCCBP groups. We found also significant positive correlation between immunoexpression of PD-L1 and the number of CD4+ cells in OSCCPP group. In conclusion, our findings support the hypothesis of involvement of Tregs and PD-L1 in OSCC development and progression.

## Introduction

Cancer of the oral cavity is the sixth most common malignancy reported worldwide [1]. It is the most common cancer in males and the third most common cancer in females [2]. Every year, an estimated 3 million new cases occur worldwide, and the overall 5-year survival rate for oral squamous cell carcinoma (OSCC) is only 50%. Chronic inflammation, coupled with alcohol, betel quid and cigarette consumption is associated with oral squamous cell carcinoma [3].

Regulatory T cells (Treg) consist of functionally diverse subsets of immunosuppressive T cells that play a crucial role in the modulation of immune responses and the reduction of deleterious immune activation [4]. Several subsets of Treg cells have been identified and characterized, such as CD8^+^ Treg cells, CD4^+^ Treg cells, and γδ-TCR [5]. Tregs can be divided into two subpopulations: natural occurring (nTreg) which develop in the thymus, and adaptive (iTreg) generated in the periphery from CD4^+^ naive T cells [6]. Tregs are capable of migrating to inflammation sites and suppressing a broad range of effector lymphocytes, particularly helper T (Th) cell subsets, such as Th1, Th2, Th17, and follicular Th (Tfh) cells [7, 8]. Until now, Foxp3 (forkhead box P3) has been the most specific marker distinguishing Treg cells from T cells. Foxp3 is a member of the forkhead/winged-helix family of transcription factors that are critically involved in the development and function of Tregs [9]. The lack of Tregs due to the loss of Foxp3 function leads to autoimmune diseases whereas high prevalence of Tregs in the peripheral blood due to the over-expression of Foxp3 causes immunodeficiency [10]. Foxp3^+^ Tregs can suppress the activation, proliferation, and effector functions of numerous cell types, including CD4^+^, CD8^+^ T cells, dendritic cells (DCs), B cells, and natural killer (NK) cells [11].

Emerging evidence supports the notion that Treg population plays a critical role in the suppression of anti-tumor immune response and thus contributes to cancer progression. Tregs can mediate peripheral tolerance by suppressing self-antigen reactive T cells [12]. Since most tumor antigens are self-antigens, the suppression of tumor antigen reactive T lymphocytes by Tregs is an important obstacle in antitumor immunity [13]. Tumor cells can secrete soluble factors that promote the induction, expansion, and recruitment of Treg cells to the tumor microenvironment. Therefore, tumors contribute to the generation and expansion of Treg cells in the tumor microenvironment [6]. There are three ways of Tregs accumulation within the tumor microenvironment: increased migration, preferential Treg cells expansion, and de novo conversion of Foxp3^−^ T cells into Treg cells [14].

The programmed death-ligand 1 (PD-L1) belongs to the B7 superfamily, which also includes B7–1 (CD80), B7–2 (CD86), B7-DC (PDL2), B7-H2, B7-H3, B7-H4 and B7-H6 [15]. PD-L1 has two known receptors, programmed death-1 (PD-1) and B7–1 (CD80) [16]. PD-1 as a dominant receptor, belongs to the CD28 family and is expressed on T cells, dendritic cells, natural killer cells, macrophages and B cells [15]. PD-L1 is constitutively expressed on murine T, B cells, DCs, macrophages, mesenchymal stem cells and cultured bone marrow-derived mast cells [17]. PD-L1 is also expressed on non-hematopoietic cells, including epithelial, vascular endothelial, muscle cells, hepatocyte, pancreatic and astrocyte cells, in addition to its expression in the eye [17], lung, kidney, spleen, thymus, placenta, and the heart [18].

PD-L1 is both inducible and constitutively expressed on cells of many solid and hematologic malignancies [19–23]. The abnormal expression of PD-L1 has been linked with prognosis and treatment response in multiple malignancies. An overexpression of PD-L1 has been observed in different solid tumors including melanoma [19], colorectal cancer [20], lung cancer [21], pancreatic carcinoma [22] and hepatocellular carcinoma [23].

Mounting evidence suggests that the PD1:PD-L1 pathway may play a central role in antigen-specific T cell response mediating PD-1-dependent immune suppression. When PD-1 interacts with cells bearing one of its ligands, which can be highly expressed on cancer cells, the ability of T cells to target the tumor cells can be effectively subverted [24]. Tumors can thereby employ the PD-1:PD-L1 inhibitory pathway to silence the immune system. Thus, interrupting this interaction can improve the ability of T cells to attack tumor cells.

Tumors escape immune surveillance by a number of mechanisms of which four groups have now been proposed on the basis of their PD-L1 status and the presence or absence of tumor-infiltrating lymphocytes (TILs). These include type I (PD-L1^pos^ with TILs driving adaptive immune resistance), type II (PD-L1 negative with no TIL indicating immune ignorance), type III (PD-L1^pos^ with no TIL indicating intrinsic induction) and type IV (PD-L1 negative with TIL indicating the role of other suppressor(s) in promoting immune tolerance) [25].

Therefore, the objectives of this study were to evaluate the immunoexpression of PD-L1 and the number of CD4^+^, CD8^+^, Foxp3^+^ cells in oral cancers. Another purpose was to find possible association between number of Foxp3^+^, CD4^+^, CD8^+^ cells and immunoexpression of PD-L1.

## Material and Methods

### Patients

Seventy eight formalin-fixed, paraffin-embedded tissue specimens of oral squamous cell carcinomas (OSCC), and eighteen control cases (normal mucosa, non-cancer affected patients) were retrieved from archival material (Chair of Pathomorphology, Medical University of Lodz, Poland). Paraffin-embedded tissue sections taken from postoperative material were diagnosed using a standard haematoxylin and eosin staining and the histological diagnoses were established according to the current standards [26]. The main criteria for patients selection was the same anatomical localization of lesions (the floor of the mouth). To find the possible relationship between the studied markers and clinical prognosis, patients with OSCC were additionally divided into two groups: with better prognosis – OSCCBP (without metastases, *n* = 37), and with poorer prognosis – OSCCPP, (with metastases to regional lymph nodes or/and with distant metastases, *n* = 41). The histopathological grade was classified into groups according to the WHO classification (for OSCCBP: G1 *n* = 3, G2 *n* = 33, G3 *n* = 1, and for OSCCPP: G1 *n* = 0, G2 *n* = 36, G3 *n* = 5). The age range for OSCCBP group was from 28 to 75 years (mean ± SD = 59,24 ± 10, 89), for OSCCPP group was from 40 to 84 (mean ± SD = 59,39 ± 11,16) and for control cases 15 to 74 (mean ± SD = 47,05 ± 18,71).

### Immunohistochemistry

Paraffin-embedded, 3-μm tissue sections were mounted onto SuperFrost slides (SuperFrost Plus, Gerhord Menzel GmbH, Braunschweig, Germany), deparaffinized in xylene and ethanol of graded concentrations. For antigen retrieval, the slides were treated in a microwave oven in a solution of TRS (Target Retrieval Solution, High pH, Dako, Denmark) for 30 min (2 × 6 minutes 360 W, 2 × 5 180 W, 2 × 4 minutes 90 W). After cooling down at room temperature, they were transferred to 0,3% hydrogen peroxide in methanol, for 30 min, to block endogenous peroxidase activities. Sections were rinsed with Tris-buffered saline (TBS, Dako, Denmark) and incubated from 30 to 60 min with monoclonal mouse primary antibodies against: CD4 (Dako; clone: 4B12, dilution 1:40), CD8 (Dako; clone: C8/144B, dilution 1:50), Foxp3 (Abcam; clone: 236A/E7, dilution 1:50), and rabbit polyclonal antibody against PD-L1 (Abcam; dilution 1:400). Immunoreactive proteins were visualized using adequate EnVision-HRP kit (Dako, Carpinteria, CA, USA) according to the instructions of the manufacturer. Visualisation was performed by incubation the sections in a solution of 3,3′-diaminobenzidine (Dako, Denmark). After washing, the sections were counterstained with Mayer’s hematoxylin and mounted. For each antibody and for each sample a negative control was processed. Negative controls were carried out by incubation in the absence of the primary antibody and always yielded negative results.

In each specimen distribution and cytoplasmic staining intensity of PD-L1 in cancer cells were recorded semiquantitatively by two independent observers in 7–10 (depending on the specimen size) adjacent high power fields and graded from 0 (staining not detectable), 1 (weak immunostaining), 2 (moderate immunostaining intensity) and 3 (strong staining). The mean grade was calculated by averaging grades assigned by the two authors and approximating the arithmetical mean to the nearest unity.

### Morphometry

Foxp3^+^, CD4^+^ and CD8^+^ cells were evaluated using computer image analysis system consisting of a PC computer equipped with a Pentagram graphic tablet, Indeo Fast card (frame grabber, true-color, real-time), produced by Indeo (Taiwan), and color TV camera Panasonic (Japan) coupled with Carl Zeiss microscope (Germany). This system was programmed (MultiScan 18.03 software, produced by Computer Scanning Systems, Poland) to calculate the number of objects (semiautomatic function).

The number of Foxp3^+^, CD4^+^ as well as CD8^+^ cells was estimated by counting all positive cells in 7–10 high power monitor fields (HPF) (0.029 mm^2^ each), marking immunopositive cells (semiautomatic function). The results were presented as a number of positive cells per HPF.

### Statistical Methods

Differences between groups were tested using unpaired Student’s t-test preceded by evaluation of normality and Levene’s test. The Mann-Whitney U test was used where appropriate. Correlation coefficients were calculated using Spearman’s method. Results were considered statistically significant if *p* < 0.05.

## Results

The cytoplasmic, perinuclear and nuclear pattern of Foxp3 immunoexpression on tumor infiltrating cells was seen in all OSCC cases and 9 control cases. Tumor cells were devoid of Foxp3 staining. CD4 and CD8 were clearly stained in the cell membrane of infiltrating cells of control and OSCC cases. The immunoexpression of PD-L1 in cancer cells was predominantly cytoplasmic although membranous expression was also noted. Membranous PD-L1 immunoexpression was predominantly detected on infiltrating cells. In our study PD-L1 was expressed on cancer cells and tumor-infiltrating lymphocytes as well as epithelial, vascular endothelial and infiltrating cells of control cases. The cytoplasmic immunoexpression of PD-L1 on cancer cells was noted in 37 of 41 cases of SCCPP group, in 25 of 37 cases of SCCBP group, and in 16 of 18 control cases.

**Fig. 1:**
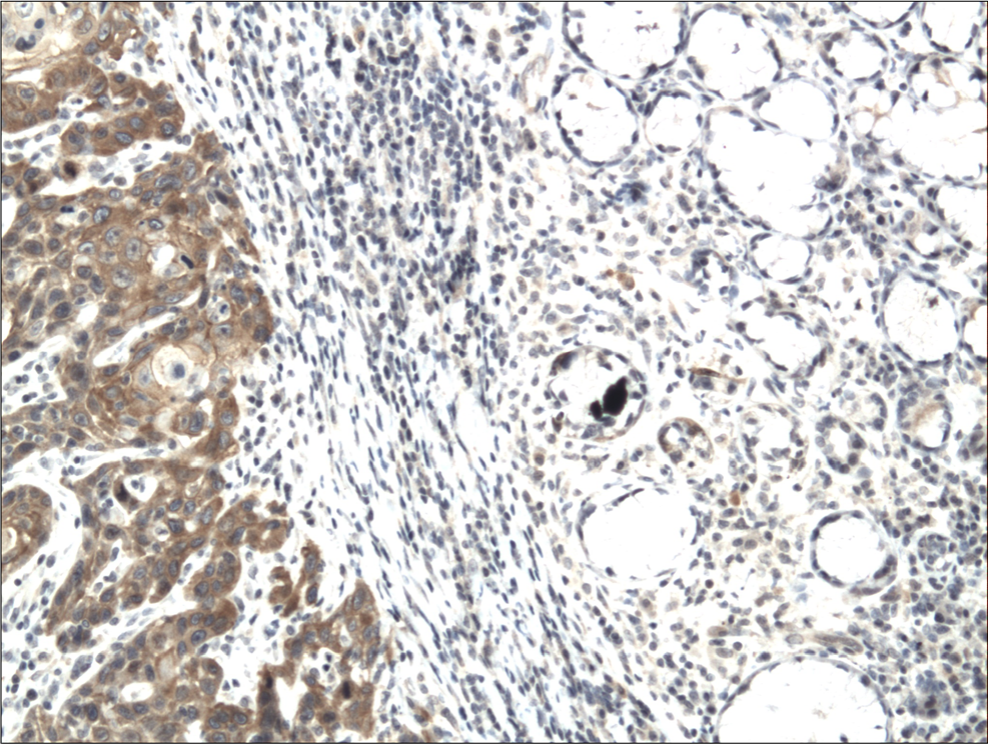
Cytoplasmic immunoexpression of PD-L1 in oral squamous cell carcinomas with poorer prognosis (OSCCPP). Immunohistochemistry. Total magnification × 100

**Fig. 2:**
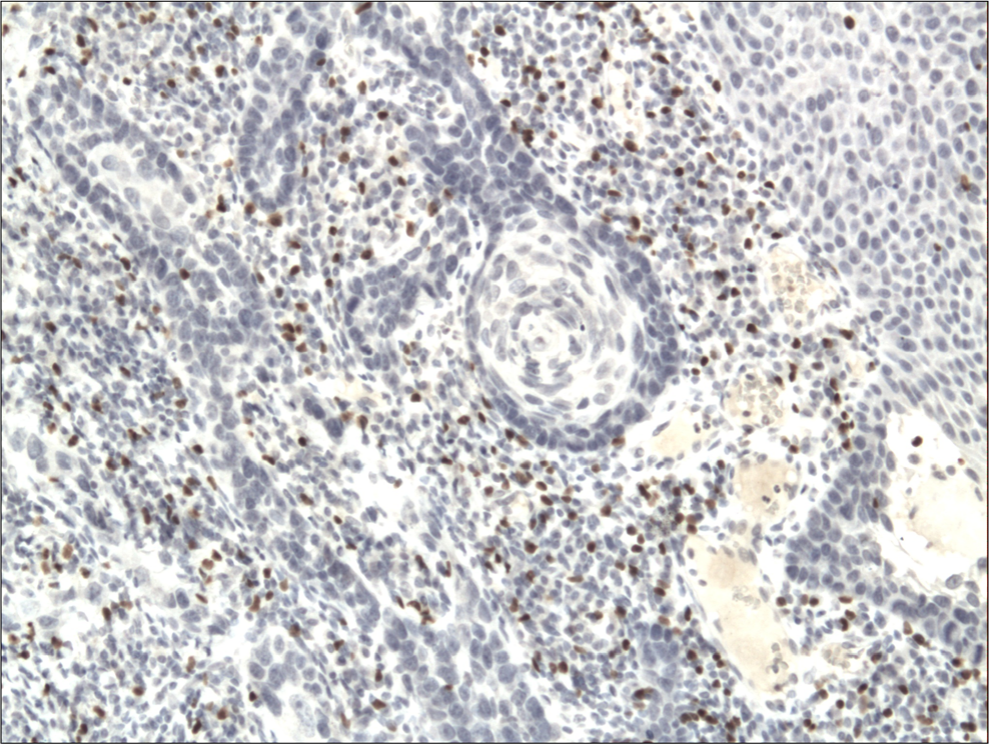
Nuclear and perinuclear immunoexpression of Foxp3 in oral squamous cell carcinomas with poorer prognosis (OSCCPP). Immunohistochemistry. Total magnification × 100

**Fig. 3:**
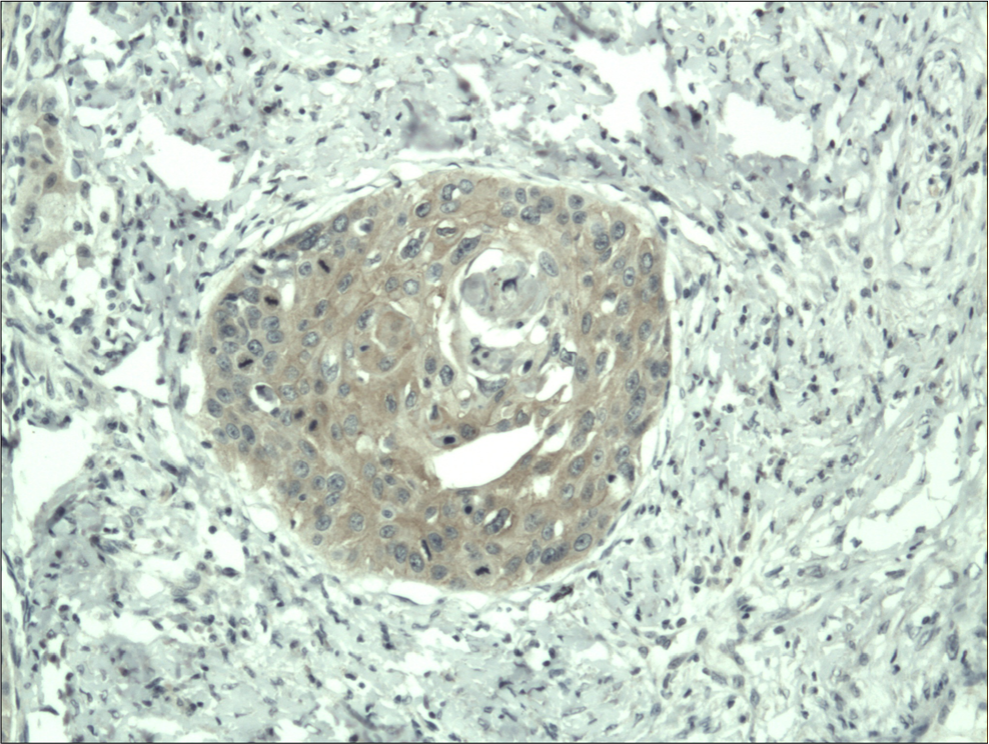
Cytoplasmic immunoexpression of PD-L1 in oral squamous cell carcinomas with better prognosis (OSCCBP). Immunohistochemistry. Total magnification × 100

**Fig. 4:**
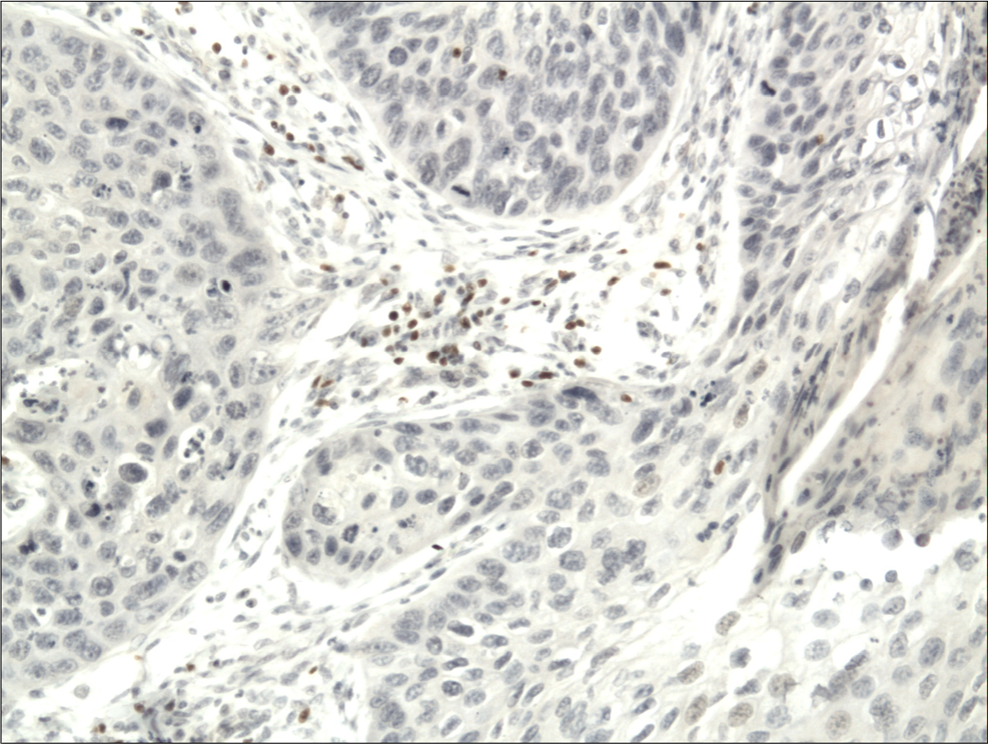
Nuclear and perinuclear immunoexpression of Foxp3 in oral squamous cell carcinomas with better prognosis (OSCCBP). Immunohistochemistry. Total magnification × 100

**Fig. 5:**
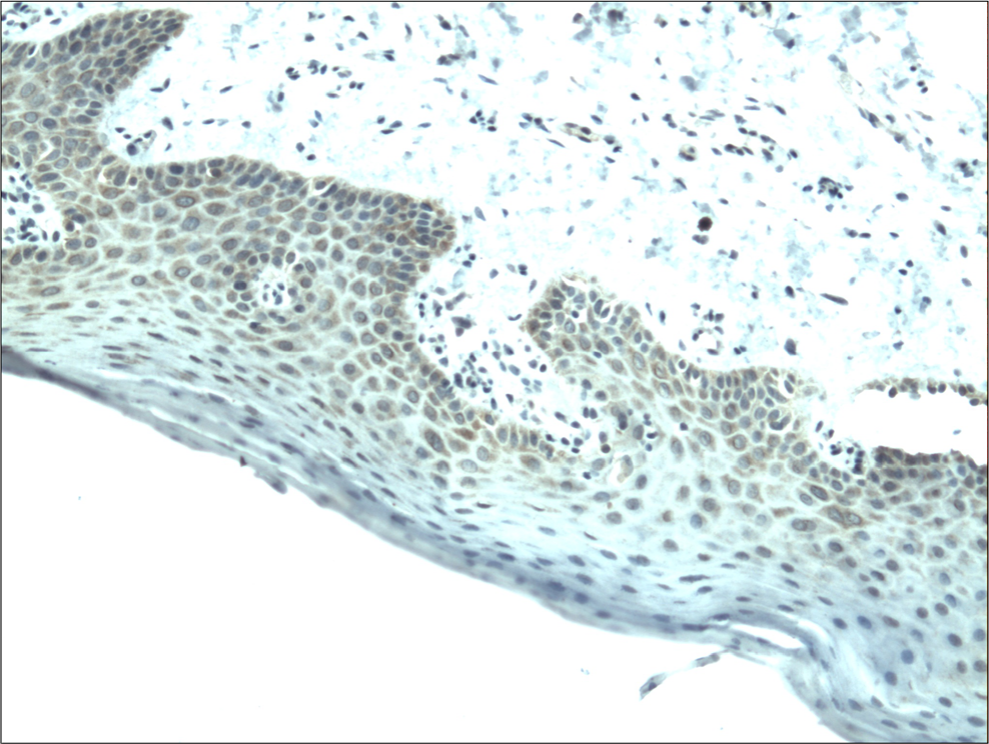
Cytoplasmic immunoexpression of PD-L1 in control. Immunohistochemistry. Total magnification × 100

**Fig. 6:**
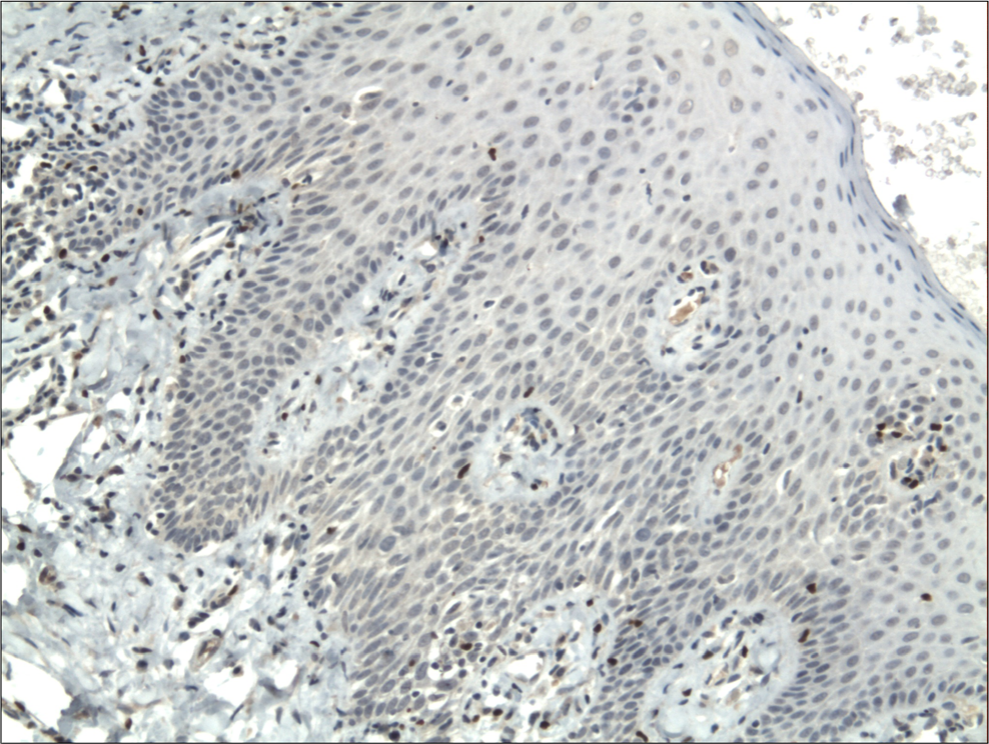
Nuclear and perinuclear immunoexpression of Foxp3 in control. Immunohistochemistry. Total magnification × 100

**Fig. 7:**
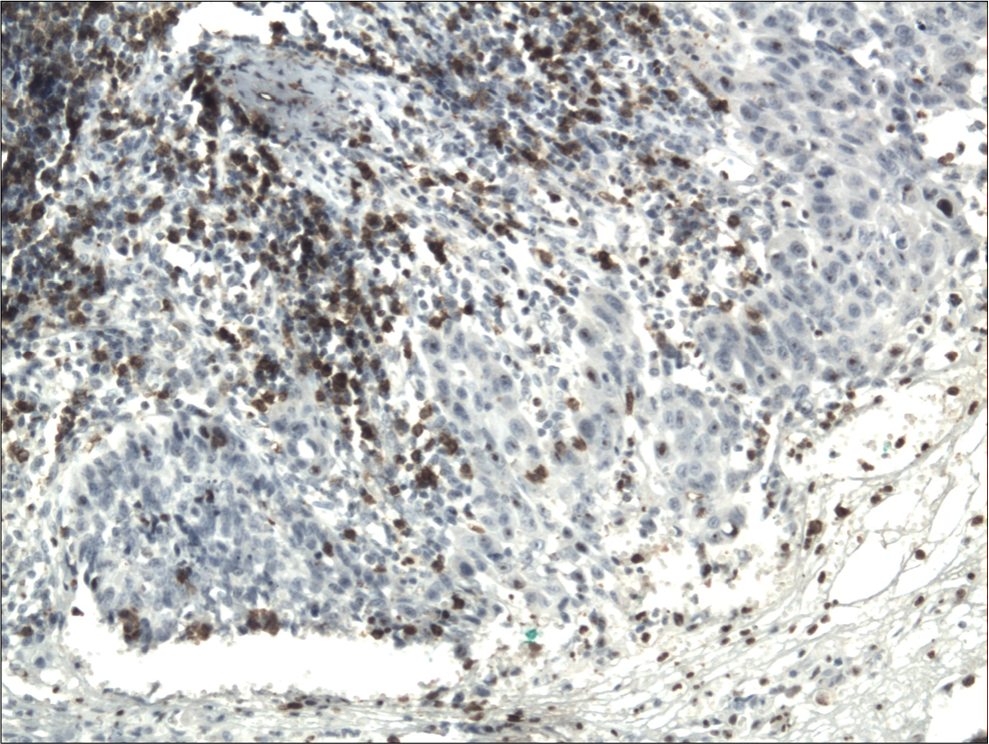
Membranous immunoexpression of CD4 in oral squamous cell carcinomas with poorer prognosis (OSCCPP). Immunohistochemistry. Total magnification × 100

**Fig. 8:**
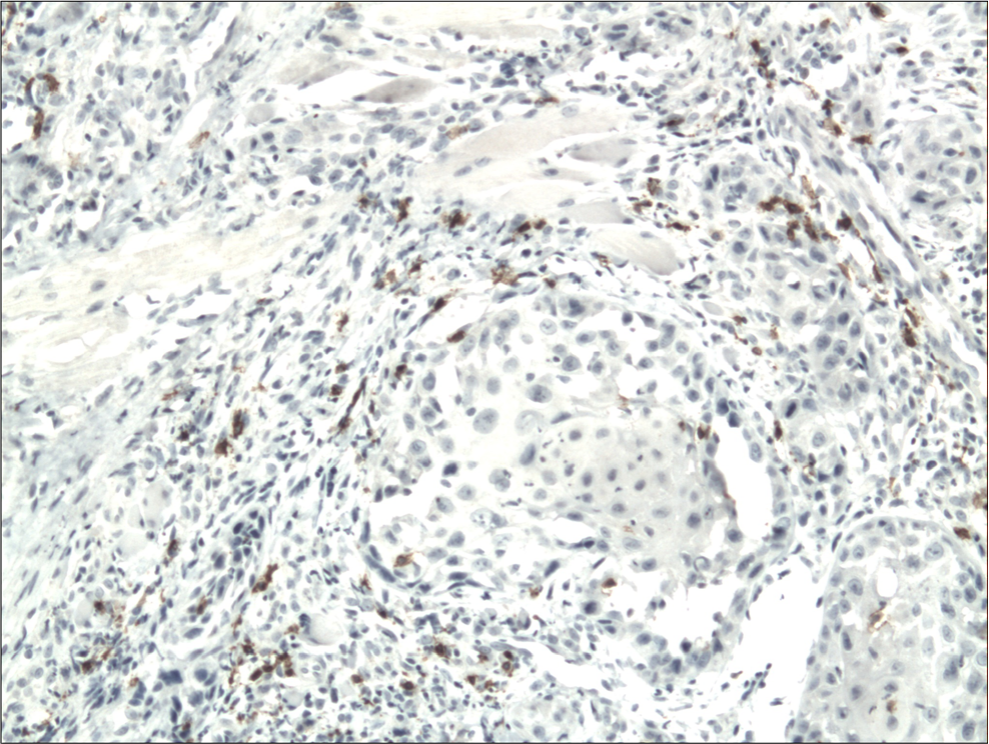
Membranous immunoexpression of CD4 in oral squamous cell carcinomas with better prognosis (OSCCBP). Immunohistochemistry. Total magnification × 100

**Fig. 9:**
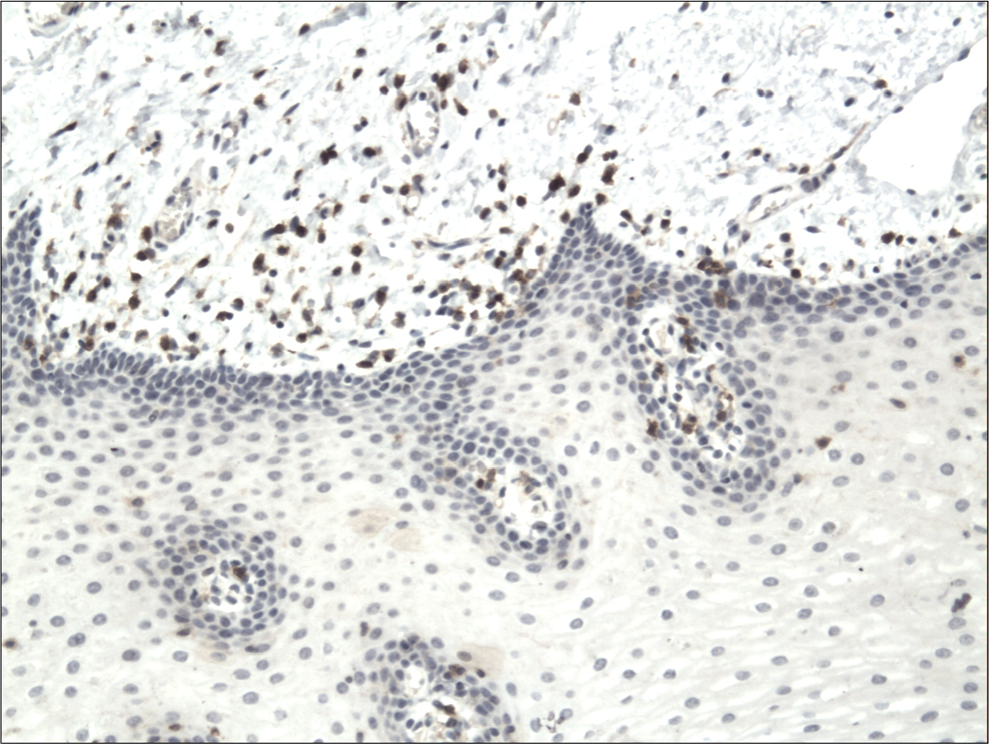
Membranous immunoexpression of CD4 in control. Immunohistochemistry. Total magnification × 100

**Fig. 10:**
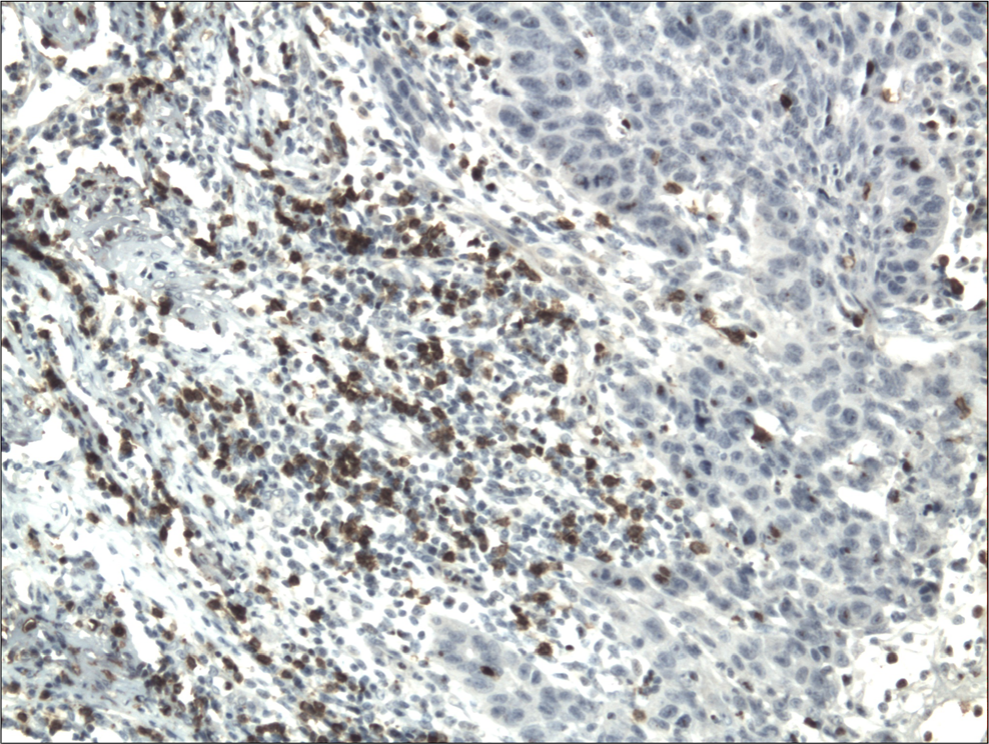
Membranous immunoexpression of CD8 in oral squamous cell carcinomas with better prognosis (OSCCBP). Immunohistochemistry. Total magnification × 100

**Fig. 11:**
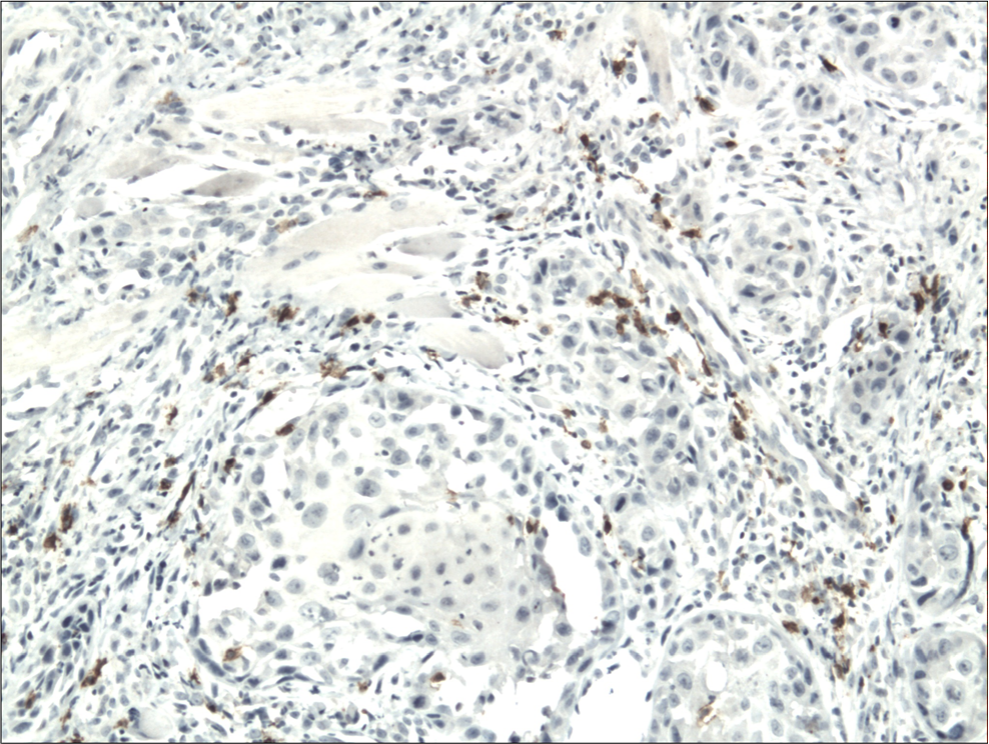
Membranous immunoexpression of CD8 in oral squamous cell carcinomas with poorer prognosis (OSCCPP). Immunohistochemistry. Total magnification × 100

**Fig. 12:**
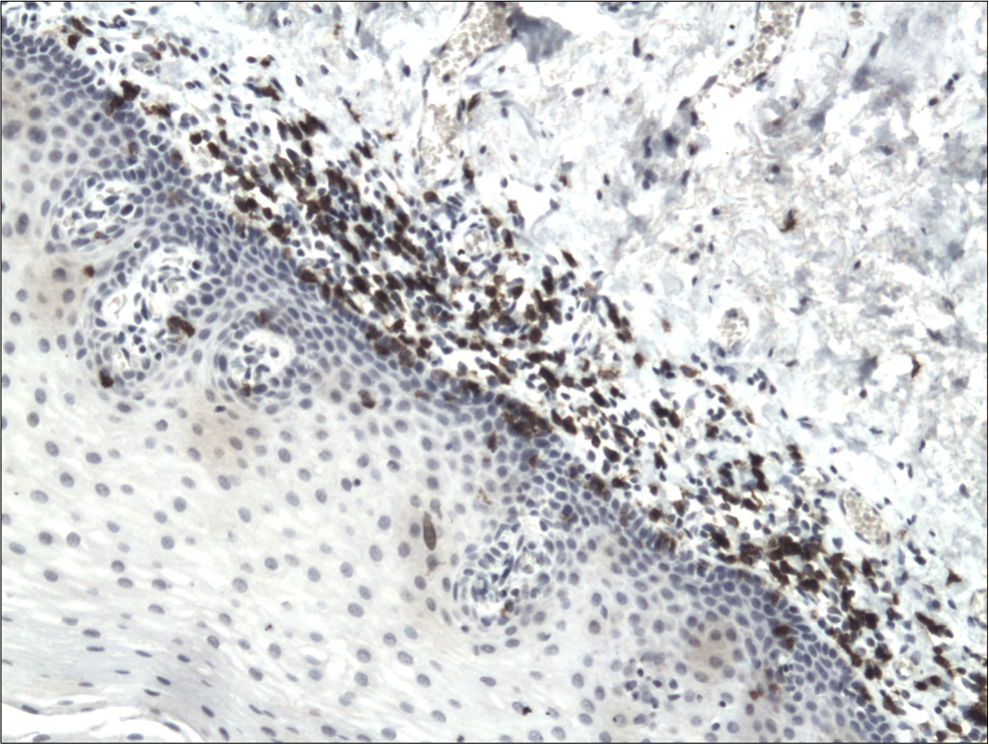
Membranous immunoexpression of CD8 in control. Immunohistochemistry. Total magnification × 100

The semiquantitative and quantitative data on the immunoexpression of PD-L1, Foxp3^+^, CD4^+^ and CD8^+^ cells appear in Table 1. The immunoexpression of PD-L1 and the mean number of Foxp3^+^ cells was significantly increased in OSCCPP group (Figs. 1 and 2), in comparison to OSCCBP (Figs. 3 and 4) and control groups (Figs. 5 and 6). We also found significantly higher immunoexpression of PD-L1 and number of Foxp3^+^ cells in OSCCBP compared to control group. The mean number of CD4^+^ cells was significantly increased in OSCCPP group (Fig. 7) in comparison to both OSCCBP (Fig. 8) and control groups (Fig. 9). CD8^+^ cells were significantly more frequent in OSCCBP group (Fig. 10) in comparison to OSCCPP (Fig. 11) and control group (Fig. 12).

**Table 1:** The immunoexpression of PD-L1, Foxp3, lymphocytes CD4+ and CD8+ in oral squamous cell carcinomas with poorer prognosis (OSCCPP), in oral squamous cell carcinomas with better prognosis (OSCCBP) and controls

Groups	PD-L1 (mean score)	Foxp3 Cells/HPF	CD4+ Cells/HPF	CD8+ Cells/HPF
OSCCPP (*n* = 41)	2.33 ± 2.02	13.4 ± 10.2	18.6 ± 12.3	9.7 ± 2.9
OSCCBP (*n* = 37)	1.32 ± 1.26	7.7 ± 6.2	12.2 ± 7.1	17.8 ± 11.1
Controls (*n* = 18)	0.63 ± 0.62	2.4 ± 1.8	9.5 ± 5.5	8.2 ± 4.3
OSCCPP vs OSCCBP	*p* < 0.011	*p* < 0.005	*p* < 0.007	*p* < 0.001
OSCCPP vs control	*p* < 0.002	*p* < 0.001	*p* < 0.004	*p* = 0.12 (NS)
OSCCBP vs control	*p* < 0.033	*p* < 0.05	*p* = 0.16 (NS)	*p* < 0.001

*NS* not significant

In both OSCCPP and OSCCBP groups there were positive significant correlations between the number of Foxp3^+^ and CD4^+^ cells, whereas the correlations between the number of Foxp3^+^ and CD8^+^ cells were not statistically significant (Table 2). The correlative study revealed in both OSCCPP and OSCCBP groups, positive correlations between the immunoexpression of PD-L1 and numbers of Foxp3^+^ cells, and negative correlation between the immunoexpression of PD-L1 and numbers of CD8^+^ cells. We found also significant positive correlation between immunoexpression of PD-L1 and the number of CD4^+^ cells in OSCCPP group (Table 3).

**Table 2:** The correlations between mean number of Foxp3+ and CD4+, CD8+ cells in oral squamous cell carcinomas with poorer prognosis (OSCCPP), and oral squamous cell carcinomas with better prognosis (OSCCBP)

Correlations	OSCCPP (*n* = 41)	OSCCBP (*n* = 37)
Foxp3+ vs CD4+ cells	*r* = 0. 44, *p* < 0.005	*r* = 0.35, *p* < 0.04
Foxp3+ vs CD8+ cells	*r* = − 0.21, *p* = 0.18 (NS)	*r* = − 0.27, *p* = 0.11(NS)

*NS* not significant

**Table 3:** The correlations between the immunoexpression of PD-L1 and mean number of Foxp3+, CD4+, CD8+ cells in oral squamous cell carcinomas with poorer prognosis (OSCCPP), and oral squamous cell carcinomas with better prognosis (OSCCBP)

Correlation between	OSCCPP (*n* = 41)	OSCCBP (*n* = 37)
PD-L1 vs Foxp3+ cells	*r* = 0.42, *p* < 0.007	*r* = 035, *p* < 0.04
PD-L1 vs CD4+ cells	*r* = 0.31, *p* < 0.002	*r* = 0.25, *p* = 0.14 (NS)
PD-L1 vs CD8+ cells	*r* = − 0.47, *p* < 0.05	*r* = − 0.33, *p* < 0.05

*NS* not significant

In control group all these correlations were weak and not significant (data not shown).

## Discussion

There is accumulating evidence that head and neck squamous cell carcinoma (HNSCC) patients display increased levels of nTreg cells with greater suppressive activity, compared to healthy controls [27, 28]. However, while some studies have linked higher Treg cells levels to worse clinical outcome in HNSCC [27], others have provided conflicting results [28].

Foxp3 is known as the most specific marker distinguishing Treg cells from T cells, and in our study Foxp3 was expressed on tumor-infiltrating lymphocytes - tumor cells were entirely negative. In contrary to above-mentioned results, Liang et al. [29] observed in tongue cancer, that Foxp3 can be expressed by both tumor cells and tumor-infiltrating lymphocytes and that tumor cells were the major cell types expressing Foxp3 (59,3% of tongue squamous cell carcinomas). Similar positive score for Foxp3 immunoexpression was observed in pancreatic cancer cells (61%) [30], and breast cancer tissues (57% and 73%) [31]. Subcellular staining of Foxp3 was heterogeneous in the present study. Different expression level and complex post-translational modification of Foxp3 may be possible reasons. Chen et al. [32] demonstrated that in Tregs, TCR-mediated post-translational modifications could mediate the regulation function, and influence the subcellular distribution of Foxp3. Authors revealed a change in the subcellular localization of Foxp3 from a more cytoplasmic/perinuclear to a nuclear expression pattern in Tregs activated with anti-CD3/anti-CD28 antibodies.

In accordance with previous studies in various malignant diseases [33, 34], we found significantly higher numbers of infiltrating Foxp3^+^ cells in both tested groups of OSCC compared with controls. Additionally, we found significantly increased number of Foxp3^+^ cells in the OSCCPP group compared to OSCCBP. An association between high intratumoral density of Foxp3^+^ cells and poorer clinical prognosis can suggest that the presence of Foxp3^+^ cells might play a role in OSCC progression. Increased Foxp3 Treg infiltration has been known to be associated with worse clinical outcomes and various poor prognostic factors in many cancers [33–35]. Suzuki et al. [36] found in colorectal cancer that the number of intratumoral Foxp3^+^ cells was positively associated with lymph node metastases. Furthermore, it has been reported that high numbers of circulating Tregs are associated with rapid tumor progression in experimental animal models of melanoma and in patients with melanoma. In these patients, the presence of Foxp3^+^ cells in primary tumor has also been associated with a higher frequency of metastases in the sentinel lymph node [37]. Even so, the association of Foxp3 expression and its impact on overall survival remains controversial. Kim et al. [38] observed that Foxp3 expression in tumor cells of colorectal cancer, but not in infiltrating Treg cells, were correlated with disease progression and poor prognosis. Moreover, literature data demonstrate that tumor infiltration by Foxp3^+^ Tregs is not always associated with a poor prognosis, but, on the contrary, can be associated with an improved prognosis in some cancer types. In colorectal cancer, high levels of infiltrating Treg cells were associated with early stage disease and improved prognosis [39, 40].

Tumor-derived CD4^+^ Treg cells have been extensively studied in many different types of cancer. It has been strongly suggested that antigen-specific CD4^+^ Tregs at tumor sites may significantly suppress immune responses, leading to immune tolerance of tumor cells. Among various CD4^+^ T cell fractions, a particular subset with CD4^+^CD25^+^Foxp3^+^ expression was previously described as regulatory T cells and was shown to mediate suppression [41]. Increased number of CD4^+^ cells and the positive correlation between number of Foxp3 and CD4^+^ cells was observed in both tested groups of OSCC, suggesting that increased immune infiltration is associated with an increased frequency of Treg cells within the infiltrate. Based on these data, we hypothesize also that the growth of OSCCs may induce the generation of CD4^+^ Treg cells. Moreover, we described significantly increased number of intratumoral CD4^+^ cells in the group of OSCCPP compared to OSCCBP and controls. An association between high intratumoral density of CD4^+^ cells and poorer clinical prognosis seems to be consistent with other findings [42]. In breast cancer, the frequency of CD4^+^CD25^+^FoxP3^+^ regulatory T cells was inversely correlated with clinical outcomes [42]. In a mouse model of human breast cancer, the depletion of CD4^+^CD25^+^ T cells was shown to reduce CD4^+^CD25^+^ T cell-mediated suppression, improve immunity, and enhance tumor regression [43].

We found significantly increased number of CD8^+^ cells in OSCCBP group in comparison to OSCCPP patients and controls. These results seem to be consistent with other findings. Zhu et al. [44] observed that breast cancer patient survival was associated with higher frequencies of CD8^+^ cytotoxic T cells in infiltrating lymphocytes. Emerging evidence from clinical studies emphasizes the role of CD8^+^ T cells in the control of tumor growth and the prolongation of patient survival [45, 46]. Lack of significant correlations between number of Foxp3^+^ and CD8^+^ cells in both studied groups of cancers suggest that CD8^+^ cells observed in our study may have other than regulatory functions or represent non-traditional subpopulation of Tregs. Recent studies demonstrated that Foxp3^+^ T cells are heterogeneous with respect to phenotype, gene expression, and function, including suppressive and non-suppressive subpopulations [47]. Miyara et al. [47] divided human Foxp3 + cells into three functional subpopulations: effector, resting and non-suppressive cytokine-secreting Tregs.

In recent years, many studies have confirmed that cancer cells can evade host immune systems by expressing certain ligands that down-regulate cytotoxic T lymphocytes through inhibitory pathways that are usually initiated by ligand-receptor interactions [48]. Currently, PD-1:PD-L1 pathway seems to be a one of major mechanism of controlling tumor immunity. In our study PD-L1 was expressed on infiltrating cells and epithelial and vascular endothelial cells in control cases. Lyford-Pike et al. [49] suggest that in normal tissue, PD-L1 is induced in response to inflammatory cytokines such as IFN-γ. This system represents a major mechanism for tissue protection in the setting of T cell-mediated inflammation. It is well established that PD-L1 expression is up-regulated in solid tumors where it can provide direct tumor protection, and reduce activity of PD-1 expressing tumor-infiltrating effector CD4 and CD8 T cells [24, 50]. We observed a significantly increased immonoexpression of PD-L1 in both tested groups of OSCC compared to controls. Overexpression of PD-L1 has been identified in several cancers, including the head and neck cancers [24, 48]. We found significantly higher immunoexpression of PD-L1 in OSCCPP compared to OSCCBP. Our results are in concordance with literature data [48, 51]. Thompson et al. [51] reported statistically significant association of PD-L1 expression with poor clinical outcome in gastric cancer. Lin et al. [52] indicated that a higher PD-L1 expression level was correlated with several clinicopathological factors, such as distant metastasis. These authors suggested also that PD-L1 immunoexpression might be associated with oral cancer development and progression. We found significant positive correlation between immunoexpression of PD-L1 in tumor cells and the number of infiltrating Foxp3^+^ cells in both tested groups of OSCC and between PD-L1 and number of CD4^+^ cells in OSCCPP group. Similar to melanoma [19], and in keeping with the proposed adaptive resistance hypothesis, in our study PD-L1 was not expressed uniformly within OSCCs, but rather at sites of lymphocyte infiltration. Our results suggest that PD-L1 immunoexpression on cancer cells is associated with Treg infiltration, and PD-L1 may be induced by an inflammatory microenvironment involving TILs.

We found significant negative correlation between immunoexpression of PD-L1 on cancer cells and number of infiltrating CD8^+^ cells in both tested groups of OSCC. In contrary to our results, Thompson demonstrated that gastric cancer patients with higher CD8^+^ T cell densities also have higher PD-L1 expression, indicating an adaptive immune resistance mechanism may be occurring. Lyford-Pike et al. [49] using quantitative RT-PCR found a significant increase in the expression of CD8 mRNA in PD-L1(+) as compared to PD-L1(−) in oropharyngael cancer. On the other hand, Tokito et al. [53] observed that lack of PD-L1 immunoexpression accompanied by increased CD8^+^ cells density was significantly associated with favourable survival in non-small cell lung cancer. Increased number of CD8^+^ cells in OSCCBP group, lack of correlation of PD-L1 with number of Foxp3^+^ cells and negative correlation between number of CD8^+^ cells and immunoexpression of PD-L1 seem to be consistent and suggest that tumor infiltrating CD8^+^ cells may have other than suppressive function.

Literature data and our results revealed the complicated interactions within the tumor microenvironment and emphasize that impact of individual types of immune cells may be highly dependent on many factors. We hypothesize that the microenvironment of a tumor is critically important in determining leukocyte phenotype and function. We speculate also that not only the number, type and localization of tumor infiltrating lymphocytes but activity (e.g. cytokine releasing patterns) of particular infiltrating cells can determine mutual relationship and has prognostic value.

Although our findings support the hypothesis of involvement of Tregs and PD-L1 in OSCC development and progression, further studies of the relationship between number and activity of immune infiltration cells and immunoexpression of PD-L1 on cancer cells are needed to better understand their role in oral carcinogenesis.
